# HLA-VBSeq: accurate HLA typing at full resolution from whole-genome sequencing data

**DOI:** 10.1186/1471-2164-16-S2-S7

**Published:** 2015-01-21

**Authors:** Naoki Nariai, Kaname Kojima, Sakae Saito, Takahiro Mimori, Yukuto Sato, Yosuke Kawai, Yumi Yamaguchi-Kabata, Jun Yasuda, Masao Nagasaki

**Affiliations:** 1 Department of Integrative Genomics, Tohoku Medical Megabank Organization, Tohoku University, 2-1 Seiryo-machi, Aoba-ku, Sendai, Miyagi, 980-8573, Japan

## Abstract

**Background:**

Human leucocyte antigen (HLA) genes play an important role in determining the outcome of organ transplantation and are linked to many human diseases. Because of the diversity and polymorphisms of HLA loci, HLA typing at high resolution is challenging even with whole-genome sequencing data.

**Results:**

We have developed a computational tool, HLA-VBSeq, to estimate the most probable HLA alleles at full (8-digit) resolution from whole-genome sequence data. HLA-VBSeq simultaneously optimizes read alignments to HLA allele sequences and abundance of reads on HLA alleles by variational Bayesian inference. We show the effectiveness of the proposed method over other methods through the analysis of predicting HLA types for HLA class I (HLA-A, -B and -C) and class II (HLA-DQA1,-DQB1 and -DRB1) loci from the simulation data of various depth of coverage, and real sequencing data of human trio samples.

**Conclusions:**

HLA-VBSeq is an efficient and accurate HLA typing method using high-throughput sequencing data without the need of primer design for HLA loci. Moreover, it does not assume any prior knowledge about HLA allele frequencies, and hence HLA-VBSeq is broadly applicable to human samples obtained from a genetically diverse population.

## Background

HLA loci on chromosome 6p21.3 are one of the most diverse and polymorphic region in the human genome, and the IMGT/HLA database release 3.15.0 currently containes 10,691 allele sequences [1]. HLA class I molecules present endogenous antigens to CD8+ (cytotoxic) T cells, whereas HLA class II molecules present exogenous antigens to CD4+ (helper) T cells [2]. HLA matching of classical class I loci (HLA-A, -B and -C) and three of class II loci (HLA-DQA1, -DQB1 and -DRB1) between a donor and patient lowers risks of acute graft-versus-host disease in unrelated haematopoietic stem cell transplantation [3] or organ transplantation [4]. Specific alleles of class I loci have been found to be associated with the rate of progression from human immunodefinciency virus type 1 (HIV-1) infection to the acquired immunodefiniency syndrome (AIDS) [5]. HLA-DR and -DQ loci have been found to be associated with autoimmune diseases, such as in type I diabetes [6], narcolepsy [7] and multiple sclerosis [8]. Hence, a method to determine HLA types accurately and conveniently is needed for both clinical practices and basic research.

Conventionally, HLA types have been determined at 2-digit resolution (e.g., A*01), which approximates the serological antigen groupings. More recently, sequence specific oligonucleotide probes (SSOP) method has been used for HLA typing at 4-digit resolution (e.g., A*01:01), which can distinguish amino acid differences [9]. Currently, targeted DNA sequencing for HLA typing [10] is the most popular approach for HLA typing over other conventional methods. Since the sequence-based approach directly determines both coding and non-coding regions, it can achieve HLA typing at 6-digit (e.g., A*01:01:01) and 8-digit (e.g., A*01:01:01:01) resolution, respectively. HLA typing at the highest resolution is desirable to distinguish existing HLA alleles from new alleles or null alleles from clinical perspective [11].

Because of recent improvement and cost reduction of the next generation sequencers (NGSs), several methods have been proposed to predict HLA types from high-throughput sequencing data. Seq2HLA [12] predicts 2-digit HLA types from RNA-Seq data, but is not designed for 4-digit HLA typing. HLAminer [13] predicts HLA types at 4-digit resolution based on the best alignment score of reads to the reference HLA allele sequences, whereas the most recently proposed PHLAT [14] predicts HLA types at 6-digit resolution based on the likelihood score considering SNP sites against reference sequences. Since there exists uncertainty in read alignments to highly homologous HLA allele sequences, accurate HLA typing at high-resolution is still challenging.

We have developed a computational method, HLA-VBSeq, to estimate HLA types effectively and accurately at 8-digit resolution from whole genome sequencing data. In the first step of the HLA-VBSeq pipeline, read sequences are aligned to the reference genomic sequences of the registered HLA allelles in the IMGT/HLA database, in which multiple hits are allowed. Then, HLA-VBSeq optimizes both read alignments to the HLA allele sequences and relative quantities of reads on HLA alleles simultaneously under a statistical framework by variational Bayesian inference. Our approach considers all the possible alignments of reads to HLA allele sequences, and calculates the marginal likelihood of data from gapped alignments of reads to the reference sequences, in which deletions and insertions as well as SNP sites are naturally considered. In our Bayesian approach, an optimal set of HLA allele sequences is estimated for accurate HLA typing. We apply HLA-VBSeq to the simulation data of 5x, 10x, 20x and 30x coverage and compare prediction performance of our method with those of PHLAT and HLAminer. We also apply HLA-VBSeq to the whole genome sequencing data of a CEU trio to show the effectiveness of the proposed method.

## Methods

### HLA-VBSeq pipeline

**Figure 1:**
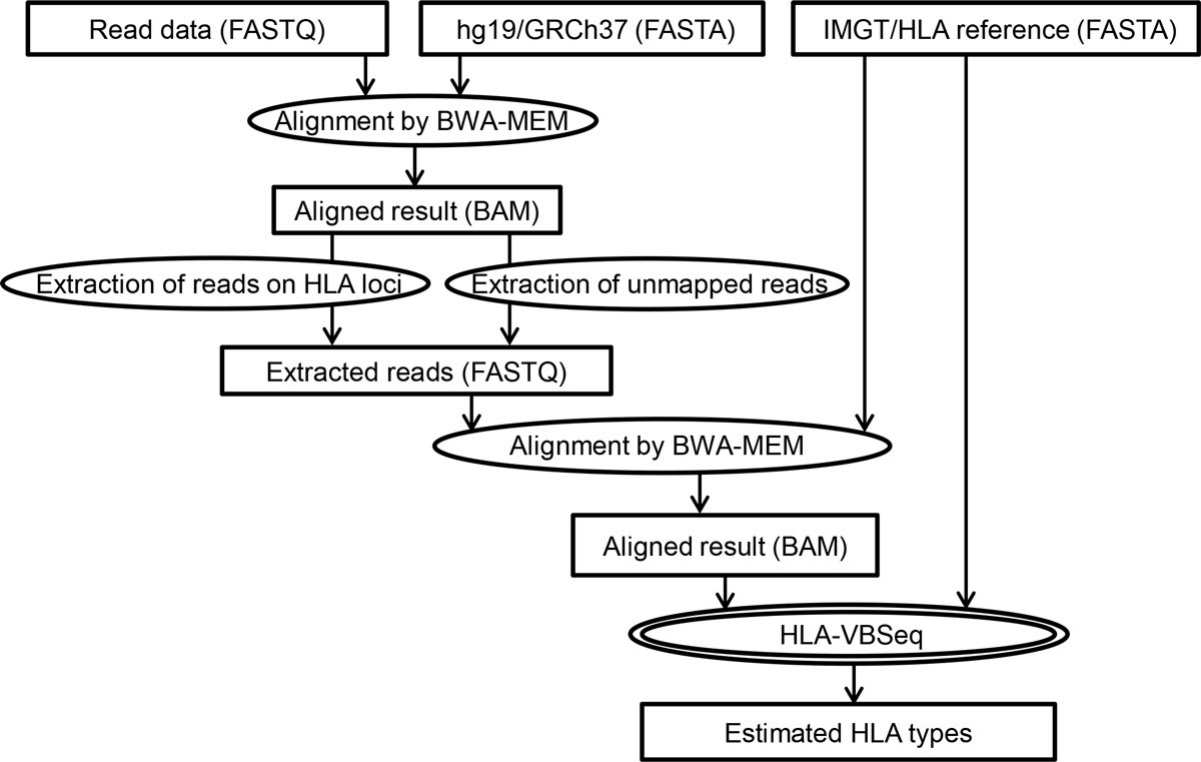
**Overview of the HLA-VBSeq pipeline**. Given read data, the human reference genome sequence, and the HLA allele reference sequences, HLA-VBSeq estimates the expected read counts on HLA alleles for HLA typing.

An overview of the HLA-VBSeq pipeline for estimating HLA types is described in Figure 1. First, reads obtained by whole-genome sequencing are aligned to the reference genome (GRCh37/hg19) with decoy sequences (hs37d5) with an alignment tool, BWA-MEM [15]. BWA-MEM is robust to sequencing errors and can be applicable to read sequence lengths up to a few megabases [16]. Second, reads aligned to HLA loci (HLA-A, -B, -C, -DM, -DO, -DP, -DQ, -DR, -E, -F, -G, -H, -J, -K, -L, -P, -V, -MIC, and -TAP) and unmapped reads are extracted from the BAM file with SAMtools [17]. For the case of paired-end sequencing data, if one of the paired-end mates is aligned to an HLA locus and the other is not, then both reads of the pair are extracted and used for downstream analyses. Then, the extracted reads are re-aligned to the collection of all the genomic HLA allele sequences in the IMGT/HLA database, in which multiple alignments to the reference sequences for each read are allowed with the "-a" option in BWA-MEM. Here, all the genomic DNA sequences registered in the IMGT/HLA database release 3.15.0, including pseudogenes, are considered. For example, the numbers of registered genomic DNA sequences for HLA-A, -B, -C, - DQA1, -DQB1, and -DRB1 are 126, 168, 118, 27, 18, and 27, respectively. Finally, expected read counts on HLA alleles are estimated by variational Bayesian inference under a statistical framework for HLA typing. Details are described in the following sections.

### Optimization of read alignments to HLA allele sequences

**Figure 2:**
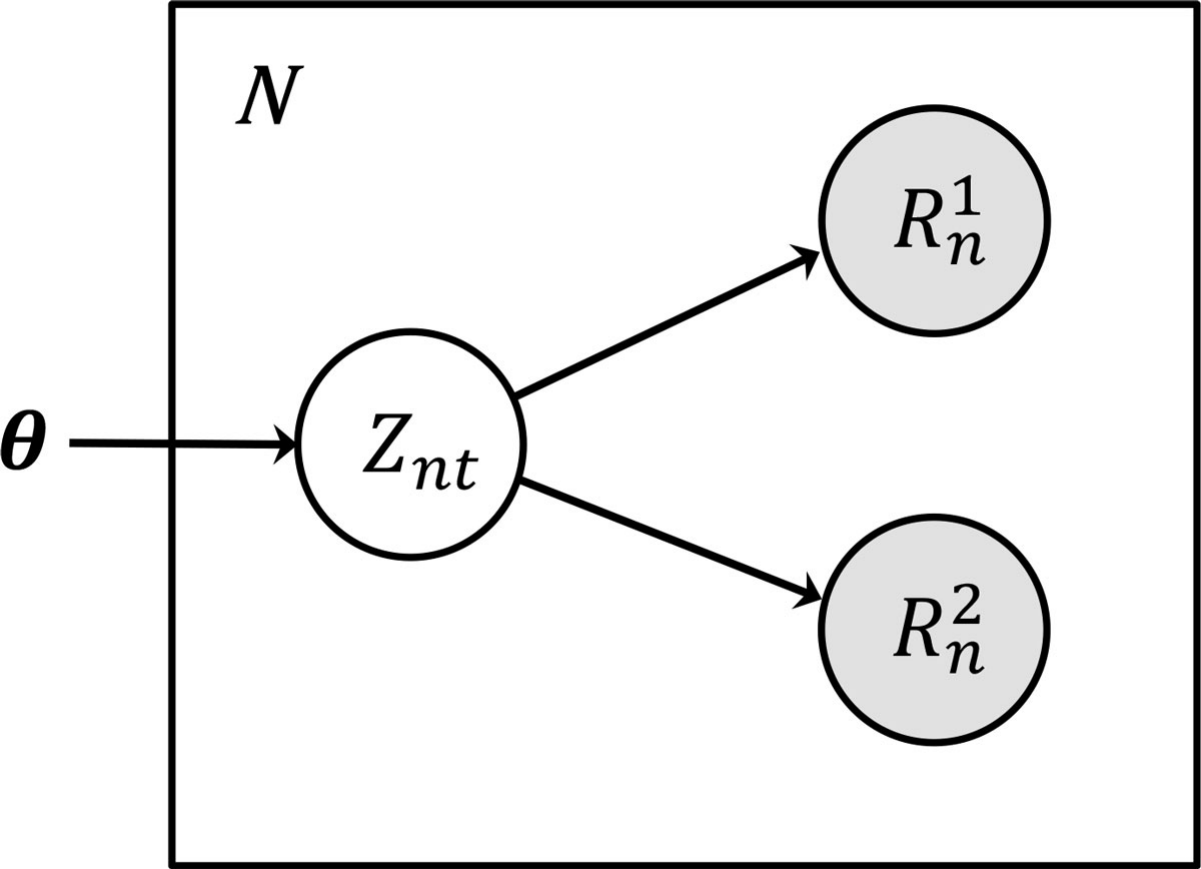
**The generative model of read data in HLA-VBSeq**. The read abundance parameter, indicator variable for the HLA allele choice, nucleotide sequence of the first and second pair of read *n *are represented by ***θ***, *Z*_*nt*_, $R_{n}^{1}$ and $R_{n}^{2}$, respectively.

From the BAM file constructed above, our goal is to estimate the most appropriate alignments of reads to the allele sequences, and to predict the most likely set of HLA types at the same time. The generative model of read data used in HLA-VBSeq is described in Figure 2. In the statistical framework, an ambiguous alignment of the first and second nucleotide sequences of read *n *to the reference HLA allele sequence *t *is treated as the hidden variable *Z*_*nt*_, where *Z*_*nt *_is an indicator variable and it takes one if read *n *is generated from allele *t*, and zero otherwise. Read abundance (depth of coverage, after normalization by the length of the allele sequence) on HLA alleles are treated as a model parameter ***θ***. In the variational Bayesian approach, model parameters are estimated as the posterior distribution. We use the Dirichlet distribution for the prior distribution of the parameter vector ***θ***

$$P\left(\theta \right)=\frac{1}{C}\overset{T}{\underset{t=0}{\prod }}\theta_{t}^{\alpha_{t}-1},$$

where *C *is a constant, $\overset{T}{\underset{t=0}{\sum }}\theta_{t}=1$, *T *is the number of HLA alleles considered, and *α*_*t *_> 0 is the hyperparameter, which controls the complexity of model parameters. In our method, we use the uniform hyperparameter *α*_0 _and it is selected as a maximizer of the log marginal likelihood of the observed data.

Our goal is to estimate the posterior distributions of ***θ ***given the data. However, this requires integrals over hidden variables and is intractable to compute in closed form. Hence, we use variational Bayesian (VB) inference to obtain the approximation of the full posterior distribution by assuming the factorization of latent variables and model parameters [18]. In the variational Bayesian E (VBE) step, for read *n*, an expected read count generated from the HLA allele *t *is calculated based on the current estimate of the abundance parameter as $\hat{r_{t}}=\underset{n}{\sum }E_{Z}\left[Z_{nt}=1\right]$. In the variational Bayesian M (VBM) step, the read abundance on each allele is calculated based on the current estimate of the expected read counts by $E_{\theta }\left[{\hat{\theta }}_{t}\right]=\hat{\alpha_{t}}/\left(\underset{t^{\prime }}{\sum }\hat{\alpha_{t^{\prime }}}\right)$, where $\hat{\alpha_{t}}=\alpha_{0}+\hat{r_{t}}$ Each step is iterated until a convergence criterion is satisfied (when the read quantities on HLA alleles are no longer updated). Update equations in each step can be calculated similarly as described in the previous work [19].

### HLA typing from the optimized read alignment on HLA alleles

After the inference algorithm converges, HLA types are predicted based on the expected number of reads assigned to each allele. Because there exist sequencing errors (substitutions, deletions and insertions against reference sequences) and alignment errors, a threshold for the depth of coverage on HLA alleles is set. In our analysis, we set the threshold as 20% of the depth of coverage (i.e., if the data is 30x on average, then we use 6x for a threhold). For each HLA locus, a diplotype is decided as follows:

• If there is no allele that passes the threshold, then it is considered that there are not enough reads to identify a correct HLA type, and hence no allele is called.

• If there is only one allele that passes the threshold, and the depth of coverage is more than or equal to twice as that of the threshold, then the HLA locus is considered to be homozygous of that HLA allele. If the depth of coverage is less than twice as that of the threshold, then the allele is called as heterozygous.

• If there are two or more alleles that pass the threshold, then alleles are sorted according to the depth of coverage from high to low. The top two alleles are selected as candidates of HLA types. If the depth of coverage of the top one is more than twice as that of the second one, then the HLA locus is set as homozygous of the top one. Otherwise, the HLA locus is predicted as a diplotype of the top and second one.

### Performance measure of HLA typing

Prediction performance is evaluated in terms of the prediction accuracy. In our analysis, the prediction accuracy is defined as the fraction of true positive predictions among the true HLA types. In this simulation experiment, two HLA alleles (either heterozygous or homozygous) for six HLA loci (HLA-A, -B, -C, -DQA1, -DQB1 and -DRB1), or 12 HLA alleles in total, are evaluated for each individual. The prediction performance is evaluated separately at the 2-digit, 4-digit, 6-digit and 8-digit resolution for each method.

## Results and discussion

### Simulation data analysis

First, we evaluate the performance of predicting HLA types with HLA-VBSeq compared to other methods in simulation data analysis. From the comparative study recently published [14], we chose PHLAT and HLAminer as comparable methods that can type HLA class I (HLA-A, -B and -C) and class II (HLA-DQA1, -DQB1 and-DRB1) loci at 4-digit resolution from whole-genome sequencing data. We prepared the simulation data of 1,000 human samples, whose HLA diplotypes for the six HLA loci were randomly chosen from the registered HLA alleles in the IMGT/HLA database release 3.15.0. Once HLA types are fixed for each individual, one SNP per 1,000 bp is incorporated in the HLA allele sequences for each individual, which is based on the average base diversity in the human genome [20]. Then, 100 bp paired-end read data (5x, 10x, 20x and 30x), whose mean and standard deviation of the fragment length distribution were set as 300 bp and 40 bp, respectively, are generated with 0.1% substitution, deletion and insertion errors uniformly across HLA allele sequences.

**Figure 3:**
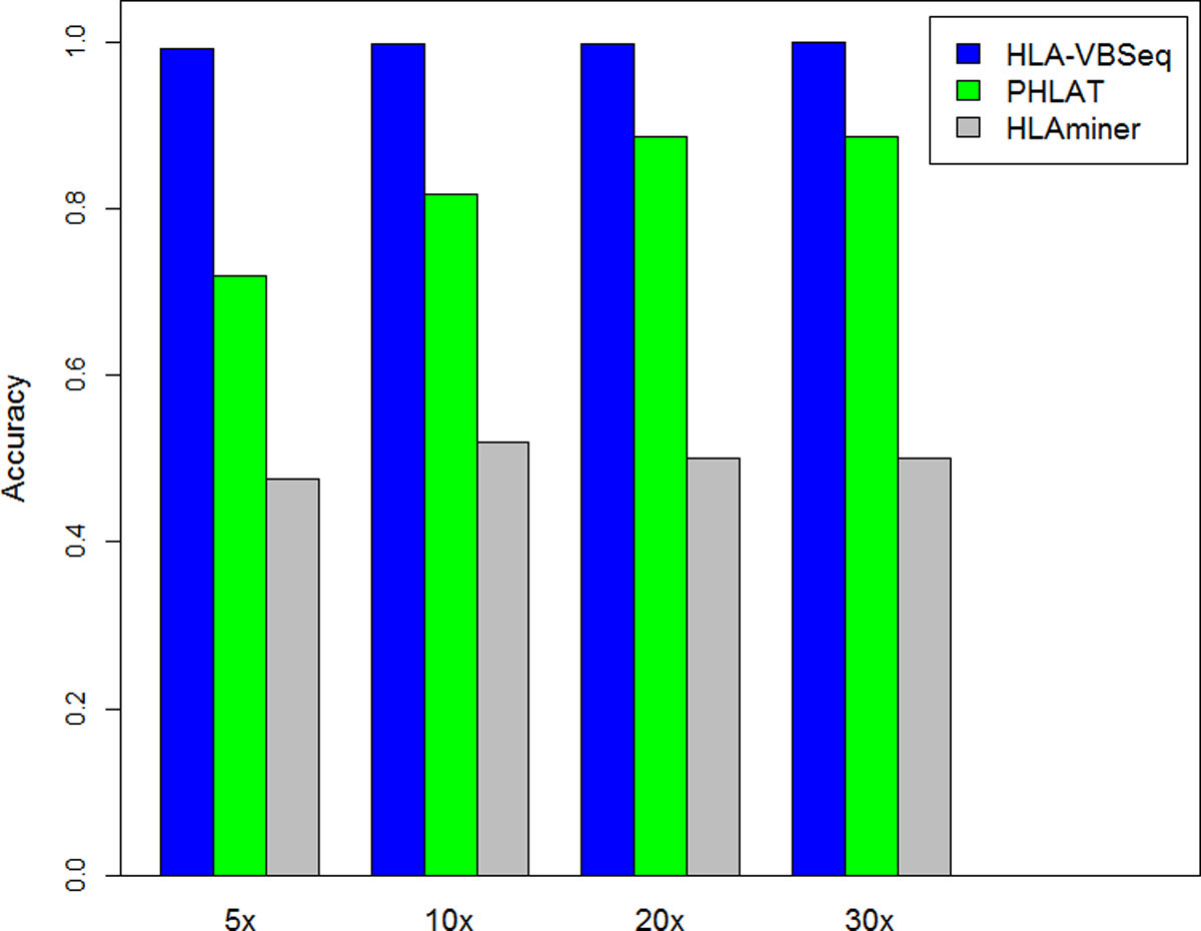
**Prediction accuracy of HLA-VBSeq and existing tools for HLA typing at 4-digit resolution for 5x, 10x, 20x and 30x simulation data**. The accuracies of each method are calculated at 4-digit resolution from 5x, 10x, 20x and 30x simulation data. HLA-VBSeq outperformed others for all the depth of coverage.

Table 1 shows the prediction accuracy of HLA-VBSeq and existing tools for HLA typing in the 30x simulation data analysis. In this experiment, all the diplotypes for the six loci were called with HLA-VBSeq. Notably, HLA-VBSeq predicts HLA types at 8-digit resolution with 99.94% accuracy, which is significantly better than those with PHLAT and HLAminer at any resolution. We did not observe a significant difference in terms of the prediction accuracy between HLA class I and class II with HLA-VBSeq (99.90% and 99.98%, respectively). Figure 3 shows the prediction accuracy of HLA typing at 4-digit resolution at the various depth of coverage of the simulation data. The prediciton accuracy with HLA-VBSeq is consistently better than those with PHLAT and HLAminar across all the depth of coverage. Notably, the HLA-VBSeq predicted HLA types at 4-digit resolution with an accuracy of 99.36% even from the 5x simulation data. Because PHLAT only considers SNP sites for calculating the likelihood, it is not effective for cases where other polymorphic sites, such as deletions or insertions, are important for determining HLA types. Another possible drawback of PHLAT is that the method require prior information about HLA allele frequencies. However, since HLA allele frequencies are diverse among human populations [21], it is not always possible to assume ethnic origins of samples.

**Table 1:** Prediction accuracy of HLA-VBSeq and existing tools for HLA typing in the 30x simulation data analysis.

HLA resolution	HLA-VBSeq	PHLAT	HLAminer
8-digit	99.94%	-	-

6-digit	99.95%	80.80%	-

4-digit	99.95%	88.75%	50.12%

2-digit	100%	96.39%	77.82%

The accuracies of each method are calculated at 8-digit, 6-digit, 4-digit, and 2-digit resolution from the 30x simulation data. PHLAT only predicts HLA types at 6-digit, 4-digit and 2-digit resolution, and HLAminer only predicts HLA types at 4-digit and 2-digit resolution.

### Real data analysis

HLA-VBSeq was applied to the PCR-free whole-genome sequencing data of CEU trio samples, NA12878, NA12891, and NA12892. The 100 bp paired-end data were generated with the HiSeq2000, whose average insert size is 300 bp and depth of coverage is 45x for each sample (all the data were generously provided by Illumina Inc.). Table 2 shows the summary of the predicted HLA types of the CEU trio. The HLA types for class I loci (HLA-A, -B, and -C) estimated with HLA-VBSeq coincided with the experimentally validated HLA types at 4-digit resolution (shown in bold text in Table 2) [10]. Notably, many of the HLA types were predicted at 8-digit resolution with HLA-VBSeq. Predicted HLA types of HLA-A, -B and -C loci with HLA-VBSeq were coincided with those with PHLAT at 6-digit resolution except B*07:02:01 (one allele in NA12891). Another literature also reported that one of HLA-B alleles of NA12891 as B*07:02:01 [22]. Instead, PHLAT predicted the corresponding HLA type as B*07:02:29. Overall, HLA types of the trio (child, father and mother) predicted with HLA-VBSeq were more consistent than those with PHLAT.

**Table 2:** Predicted HLA types of the CEU trio samples with HLA-VBSeq.

Sample	Predicted HLA types	
NA12878 (child)	A*01:01:01:01	A*11:01:01
	B*08:01:01	B*56:01:01
	C*01:02:01	C*07:01:01:01
	DQA1*01:01:02	DQA1*05:01:01:02
	DQB1*02:01:01	DQB1*05:01:01:02
	DRB1*03:01:01:01	DRB1*01:01:01

NA12891 (father)	A*01:01:01:01	A*24:02:01:01
	B*07:02:01	B*08:01:01
	C*07:01:01:01	C*07:02:01:03
	DQA1*01:02:01:01	DQA1*05:01:01:02
	DQB1*02:01:01	DQB1*06:02:01
	DRB1*03:01:01:01	DRB1*15:01:01:02

NA12892 (mother)	A*02:01:01:01	A*11:01:01
	B*15:01:01:01	B*56:01:01
	C*01:02:01	C*04:01:01:01
	DQA1*01:01:02	DQA1*01:01:02
	DQB1*05:01:01:02	DQB1*05:01:01:01
	DRB1*01:01:01	DRB1*01:01:01

Predicted HLA types of HLA-A, -B, and -C loci with HLA-VBSeq were experimentally validated at 4-digit resolution in [10], which is shown in bold text. Predicted HLA types of HLA-DQA1, -DQB1 and -DRB1 loci with HLA-VBSeq were coincided with those with PHLAT at 6-digit resolution except DQA1*01:01:02 (one allele in NA12878 and two alleles in NA12892). PHLAT instead predicted them as DQA1*01:01:01, whose genomic sequence was missing in the IMGT database release 3.15.0., and hence HLA-VBSeq could not predict the HLA allele in our experimental condition.

Predicted HLA types of HLA-DQA1, -DQB1 and -DRB1 loci with HLA-VBSeq were coincided with those with PHLAT at 6-digit resolution except DQA1*01:01:02 (one allele in NA12878 and two alleles in NA12892). PHLAT instead predicted them as DQA1*01:01:01, whose genomic sequence was missing in the IMGT database release 3.15.0, and hence HLA-VBSeq could not predict the HLA type in our experimental condition.

## Conclusions

HLA-VBSeq is an efficient and accurate HLA typing method using whole-genome sequencing data without the need of primer design for HLA loci or prior knowledge of HLA allele frequencies. Although we have evaluated the prediction performance with HLA-VBSeq using the simulation data of various depth of coverage and real data of whole-genome sequencing data, other high-throughput sequencing data, such as from target-sequencing can be utilized with minor modifications in the pipeline. However, we should bear in mind that because of the complexity of HLA loci and a fairly polymorphic nature of each locus, off-target sequences are often obtained by the target-sequencing approach, such as from pseudogenes [23]. As population-wide sequencing data becomes available, HLA-VBSeq can be easily applied for HLA typing at any HLA loci, which will be useful for association studies to identify links to phenotypes, as well as for clinical works such as donor-recipient matching. Since the genomic sequences registered in the IMGT/HLA database are still not complete, there is room for improvement in predicting HLA types with HLA-VBSeq in the future.

## Availability of supporting data

The implementation of HLA-VBSeq and the documentation is available in the website, http://nagasakilab.csml.org/hla

## Competing interests

The authors declare that they have no competing interests.

## Authors' contributions

NN and MN conceived the study, NN, KK, and MN designed the computational experiments, NN performed the analysis, and NN, KK, TM and MN interpreted the results. SS, YS, YK, YYK, and JY collaborated on data collection and interpretation of the results. NN, KK, TM, YS, YK, YYK, JY and MN wrote the manuscript. All the authors read and approved the final manuscript.

## Declarations

The publication costs for this article were partly funded by MEXT Tohoku Medical Megabank Project.

This article has been published as part of *BMC Genomics *Volume 16 Supplement 2, 2015: Selected articles from the Thirteenth Asia Pacific Bioinformatics Conference (APBC 2015): Genomics. The full contents of the supplement are available online at http://www.biomedcentral.com/bmcgenomics/supplements/16/S2
